# Beyond the Knife—Reviewing the Interplay of Psychosocial Factors and Peripheral Nerve Lesions

**DOI:** 10.3390/jpm11111200

**Published:** 2021-11-13

**Authors:** Johannes C. Heinzel, Lucy F. Dadun, Cosima Prahm, Natalie Winter, Michael Bressler, Henrik Lauer, Jana Ritter, Adrien Daigeler, Jonas Kolbenschlag

**Affiliations:** 1 Department of Hand-, Plastic, Reconstructive and Burn Surgery, BG Klinik Tuebingen, University of Tuebingen, Schnarrenbergstraße 95, 72076 Tuebingen, Germany; lucy-felice.dadun@student.uni-tuebingen.de (L.F.D.); cprahm@bgu-tuebingen.de (C.P.); mbressler@bgu-tuebingen.de (M.B.); hlauer@bgu-tuebingen.de (H.L.); jana.ritter@comdesign24.de (J.R.); adaigeler@bgu-tuebingen.de (A.D.); jkolbenschlag@bgu-tuebingen.de (J.K.); 2 Department of Neurology, Hertie Institute for Clinical Brain Research (HIH), University of Tuebingen, Hoppe-Seyler-Str. 3, 72076 Tuebingen, Germany; natalie.winter@med.uni-tuebingen.de

**Keywords:** peripheral nerve injury, psychosocial factor, nerve repair, neuroma, brachial plexus injury, compression neuropathy, pain, depression, disability, quality of life

## Abstract

Peripheral nerve injuries are a common clinical problem. They not only affect the physical capabilities of the injured person due to loss of motor or sensory function but also have a significant impact on psychosocial aspects of life. The aim of this work is to review the interplay of psychosocial factors and peripheral nerve lesions. By reviewing the published literature, we identified several factors to be heavily influenced by peripheral nerve lesions. In addition to psychological factors like pain, depression, catastrophizing and stress, social factors like employment status and worker’s compensation status could be identified to be influenced by peripheral nerve lesions as well as serving as predictors of functional outcome themselves, respectively. This work sheds a light not only on the impact of peripheral nerve lesions on psychosocial aspects of life, but also on the prognostic values of these factors of functional outcome. Interdisciplinary, individualized treatment of patients is required to identify patient at risk for adverse outcomes and provide them with emotional support when adapting to their new life situation.

## 1. Introduction

Peripheral nerve injuries (PNIs) have annual incidence rates in the USA of 43.8/1,000,000 for PNIs of the upper extremities [1] and 13.3/1,000,000 of the lower extremities, respectively [2]. PNIs often have disastrous sequalae on the affected patients’ qualities of life, especially in case of major nerve injuries and often required long and elaborate rehabilitation [3]. Therefore, surgical treatment options to restore sensibility and muscular function as well as to relieve pain are subject of a plethora of clinical and preclinical research efforts [4,5,6,7,8]. In addition to research efforts to deepen under understanding of the peripheral nervous systems anatomy, as well as the neurobiological and pathophysiological aspects of PNIs [9], novel treatment options and concepts are rapidly evolving in the wake of refined microsurgical techniques [10]. In addition to advances in neurobiological, anatomical and (micro)surgical aspects, the current body of knowledge regarding epidemiology of PNIs is also steadily evolving. About 25 years ago, Noble was one of the first to publish epidemiological data concerning PNI in patients with multiple injuries seen at a regional Level 1 trauma center [11] and in recent years, large multicenter studies have contributed to our knowledge and understanding of the epidemiology of lower and upper extremity PNIs [12,13]. Representative patients’ characteristics and socioeconomic sequelae of PNIs have recently been described by Bergmeister and colleagues, approaching another important perspective of research regarding surgical treatment of peripheral nerve lesions. According to their observations in a study sample of 250 patients with 268 PNIs, acute in-patient treatment costs for upper extremity nerve damages ranged between 2650€ and 5000€ [14]. Although the overall incidence of PNI has decreased in recent year, treatment costs are steadily increasing [1,2]. 

The severity of PNIs and postoperative functional outcomes are usually assessed by quantitative biomedical measures and postoperative evaluations of strength, sensory function, and range of motion [15,16]. However, research shining a light on the psychosocial aspects of such injuries is yet sparse [17,18]. Psychosocial factors have been defined as “characteristics or facets that influence an individual psychologically and/or socially” by Thomas [19]. Their role has been extensively studied and reviewed in diseases and conditions such as extremity trauma [20,21] or isolated hand injuries [22,23,24]. It was also shown that negative emotions prior to surgery are associated with adverse pain outcomes and postoperative disability [25,26,27,28,29]. Besides psychological aspects, the social impact of upper extremity injuries, e.g., predictors of return to work in patients suffering from work-related injuries has been addressed before in regard to other trauma, e.g., hand lesions [30,31]. It has also been demonstrated that the ability to return to work after traumatic injury does not only depend on physical capability and health but also by other aspects including psychosocial factors like psychological distress, personal income, the educational level of the injured person or the presence of a strong social network [32,33,34]. There is yet no comprehensive review summarizing the interplay of psychosocial factors with peripheral nerve injury and functional outcome following surgical treatment of such lesions. Therefore, it is the aim of this work to compile the current body of knowledge to provide surgeons, hand therapists, occupational therapists and other disciplines involved in the treatment of peripheral nerve injuries with another perspective on psychosocial aspects of peripheral nerve injuries to enable an even more holistic treatment approach.

## 2. Methods

To gather eligible studies, a literature search on PubMed was performed using the search terms “peripheral nerve”; “peripheral nerve injuries”; “psychosocial “and “psychological”. This yielded 95 results. After removal of duplicates, 90 papers remained for eligibility screening. Both original work and review papers were deemed fitting for discussion in this work. After removal of studies and reviews which addressed topics not related to our research questions and further reference screening of eligible work, 30 papers remained to be included in this review. 

## 3. The Psychosocial Impact of Peripheral Nerve Lesions

Uvarov was among the first to publish his hypothesis that negative emotions are involved in the pathogenesis of peripheral nervous system disorders in Russian in the year 1971 [35]. However, the topic remained of little interest for the scientific community in the following years and decades. More than thirty years after Uvarov published his work, Jaquet et al. conducted a retrospective chart review of 107 patients with either median nerve injury, ulnar nerve injury, or combined injuries of both nerves. 94% of these patients experienced early psychological stress following nerve trauma. More than a third of the affected patients reported psychological symptoms of clinical depression. The authors found that combined nerve injuries had a significantly higher risk for early psychological stress following trauma when compared to isolated injuries of either the median or ulnar nerve. Severe psychological distress as assessed by means of the Impact of Event Scale (IES) was associated with more severe functional deficits, mean time off work and motor recovery. Additionally, patients with higher psychological stress had an Odds ratio of 3.32 for long-term incapacity for work. A higher education level was identified as a protective factor in regard to psychological distress following nerve injury [36]. Ultee’s group reproduced these findings in a prospective study which evaluated early posttraumatic psychological stress in 61 patients with either isolated or combined injuries of the median and ulnar nerve. The aforementioned authors found that >90% of patients experienced psychological stress one month postoperatively and about a fourth required psychological treatment based on their IES scores. Three months postoperatively, psychological stress was still reported by more than 83% of the patients, and around 13% qualified for psychological treatment. The authors identified a correlation of female gender, adult age, and combined nerve injuries with the occurrence of psychological stress symptoms 1 month postoperatively [37]. In regard to the impact of single or combined nerve injuries on the affected patients’ work lives, Bruyns and colleagues analyzed potential predictors for return to work in 81 patients with median and/or ulnar nerve injuries in a retrospective study. Within a year after injury 59% of the patients returned to work after a mean time of work of 31 weeks. While 8 out of 10 patients with median nerve lesions were able to resume their work, this applied to less than 6 out of 10 and less than 3 out of 10 patients in the groups with ulnar nerve and combined nerve lesions, respectively. The ability to return to work (RTW) was significantly associated with the educational status of the participants and high rates of RTW were found in patients with white-collar employment, in comparison to those with blue-collar employment. Compliance to hand therapy was also found to result in odds ratio of 3.5 for RTW following nerve injury and repair [34]. Novak’s group studied 158 patients who suffered from peripheral nerve injuries in the upper extremity and evaluated potential biomedical and psychosocial factors which correlated with disability. They found that patients who received worker’s compensation, were involved in litigation or who were unemployed had significantly more severe disabilities of the affected extremity than their counterparts. These disabilities which were assessed by the Disabilities of Arm, Shoulder and Hand Questionnaire (DASH) score showed a significant correlation with the level of pain in these patients. In regard to potential predictors for post-injury disabilities, pain intensity, cold sensitivity, pain catastrophizing score, depression, employment status, worker’s compensation and potential ongoing litigation were identified [18]. To summarize all aforementioned authors’ findings, patients with combined median and ulnar nerve injuries are at higher risk for psychological stress and also not return to work as compared to patients with single site nerve lesions. Additionally, patients who report high levels of injury-related neuropathic pain, suffer from depression, are unemployed or have an ongoing lawsuit are also at higher risk for post-injury disability.

Besides the number of injured nerves, another point to consider in regard to the psychosocial impact is the severity of the respective lesion. In 2009, Bailey et al. aimed to evaluate the relationship between the degree of nerve damage in the upper extremity and psychosocial parameters such as activity participation, perceived quality of life (QoL), pain, and depression. Two individual groups were analyzed, one with compression neuropathies (*n* = 25), the other suffering from traumatic PNI of the upper extremity (*n* = 24). The authors observed that their study cohort of 49 individuals have given up about a fifth of their daily activities prior to their initial surgeon consultation and a significantly greater activity loss was observable in the group with traumatic PNI. Especially high-demand leisure activity such as physical exercise was reduced by almost 50% in the nerve injury group while social activities were reduced to 82%. In the group with compression neuropathies, leisure activities decreased by 30% and social activities were reduced to 87%, respectively. Pain intensity was reported as moderate among the studied patient sample with no significant differences between groups. The overall ratings for physical and psychological qualities of life were under average and almost 40% of the studied patients suffered from signs of depression secondary to the nerve damage. Interestingly, the cut-off for clinical depression was transgressed in the group with traumatic nerve injury only, but this difference was not statistically significant. Correlation analysis revealed a strong association between activity loss on the one hand and higher levels of depression as well lower perceived QoL on the other hand. Higher level of depression did also strongly correlate with lower perceived QoL. More severe pain was moderately associated with higher depression scores but only a weak correlation was found between the former and QoL. Interestingly, no correlation was observable between pain severity and physical QoL following peripheral nerve damage. In summary, more than 60% of the observed variance could be predicted by means of the two factors depression and activity participation, indicating that these two factors could be addressed as potential targets to improve the QoL in patients with upper extremity nerve damage, regardless of its genesis. However, it must be noted that the authors did not assess the manifestation of depression or other psychosocial factors prior to nerve damage, a potential confounder for the observed results [38]. Aiming to further differentiate the psychosocial impact of different types of nerve injuries, e.g., single site compression neuropathies from more complex pathologies like brachial plexus injuries and neuromas, Mackinnon’s group performed a retrospective chart review of 490 patients presenting to their department between 2010 and 2012. The divided their patient sample into seven groups in accordance with their respective diagnosis: 1. Brachial plexus injuries; 2. Thoracic outlet syndrome (TOS); 3. single compression neuropathy; 4. dual compression neuropathy; 5. ulnar nerve lesions other than cubital tunnel syndrome; 6. compression mononeuropathy in the lower extremity and 7. neuroma. To distinguish between motor deficits due to compression of the common peroneal nerve and sensory disturbances caused by compressions of the cutaneus branches or the superficial or deep peroneal nerve, the sixth group was further subdivided. The authors reported a statistically significant difference regarding the pain-related decrease of QoL which was more severe in patients suffering from TOS, neuroma, or compression neuropathies of the superficial and deep branch of the peroneal nerve when compared to patients suffering from compression mononeuropathy in the upper extremity. Patients in the neuroma subgroup also reported significantly more stress at home as well as at work when compared to the patients with single compression mononeuropathies. Patients with dual compression neuropathies had reported higher stress levels and a decreased ability to cope with stress at work. Other factors which were identified as negative predictors for a significant decrease in QoL were female sex, smoking and anti-alcoholism. Female sex and anti-alcoholism were also associated with higher pain intensities. In regard to the reported stress-at-home, significantly higher levels were reported by female patients. Non-alcoholics had an increased risk for reduced coping abilities and higher stress-at-work levels [17]. Stonner, Mackinnon and Kaskutas conducted a retrospective cross-sectional study, including 627 patients with nerve disorders of the upper extremity. In the style of the aforementioned study conducted by Wojtkiewicz [17] patients were grouped based on the nerve disorder they were diagnosed with. The seven groups were categorized as follows: 1. lesions of the median nerve; 2. lesions of the ulnar nerve; 3. lesions of the radial nerve; 4. proximal lesions of either the axillary, long thoracic, suprascapular, or musculocutaneous nerve; 5. compression neuropathies of at least two different nerves; 6. TOS; 7. brachial plexus injuries. The authors found little difference in regard to post-injury work status when comparing the seven different groups with the exception of patients suffering from brachial plexus injuries. In this group only a fourth of the patients continued to work in their respective jobs on a daily basis and almost half of all patients did not work at all. More than ten percent of the entire study population of 627 received worker’s compensation and more than half of these patients did not return to work. These participants reported significantly higher levels of depression and higher stress at home when compared to patients which did not receive worker’s compensation and were not working. The latter group’s proportion was also markedly smaller, comprising only 14% of the patients which did not receive worker’s compensation. In regard to the overall disability following peripheral nerve lesions as assessed by the DASH score, the studied patients reported a significantly higher degree of disability in comparison to the general population. A quarter of all patients were unable to work as they wanted to. Both general disability as well as work-related disability were more severe in patient who were unemployed, received worker’s compensation or reported depression ratings which were significantly higher than the general population’s mean. While patients with brachial plexus lesions scored highest in the DASH, median nerve lesions were associated with lowest disability ratings. Both the mean mental and physical QoL were significantly lower in the participants when compared to the general population and lowest scores were observed in the patients with brachial plexus injuries. Poorer QoL ratings were associated with female sex, unemployment, worker’s compensation status and above-mean depression ratings. Final stepwise linear regression analysis yielded ten variables which accounted for more than 60% of the observed variability in reported disability ratings. Among them were 7 psychosocial factors: 1. depression; 2. the level of pain; 3. momentary unemployment; 4. difficulties in sleeping; 5. affection of intimate relationships; 6. modified job demands and 7. stress at work. 46% of variance regarding work disability could be predicted by five variables: 1. affection of intimate relationships; 2. performance of household chores by others; 3. performance of a reduced amount of household chores; 4. difficulties with sleeping and 5. performance of the same level of household chores but with pain. Around 50% of physical QoL scores were predictable by: 1. DASH score; 2. the level of pain; 3. number of medications and 4. Work DASH scores. Slightly less than 50% of variability in mental QoL scores could be explained by: 1. the ability to cope with stress at home; 2. DASH score; 3. stress at home and 4. sleeping difficulties [39]. In accordance with the aforementioned authors’ findings, Yannascoli et al. found significantly increased rates of coded depression and coded anxiety in their study sample of >1800 patients suffering from brachial plexus injury as compared to >18,000 healthy subjects. While 46% of the control patients had coded depression and/or anxiety, this rate was 54% in the group of patients suffering from brachial plexus injuries. Additionally, there were significantly increased incidences of new-onset postoperative depression (20%) and anxiety (12%) in the latter group when compared to the healthy controls [40]. Landers et al. reported that about one fifth of their study population of 21 patients with brachial plexus injuries met criteria for posttraumatic stress disorder (PTSD) and exhibited clinical depression, respectively. Most concerningly, about a third of the studied patients reported suicidal ideation [41]. In regard to the impact of injury severity in case of isolated hand injuries, Tezel’s group found no correlation between psychological morbidities and injury severity and hand function, respectively in patients suffering from traumatic hand injury with major nerve involvement. The hand injury severity as assessed by the modified Hand Injury Severity Score (MHISS) correlated significantly with the patients’ ability to return to work following hand trauma [42]. In conclusion, the reviewed studies indicate, that traumatic nerve injuries are more likely to have a strong psychosocial impact as compared to compression mononeuropathies. Notably, severe nerve lesions, especially brachial plexus injuries, have devastating consequences for the affected patients both in regard to employment status and work life as well as mental health [43]. 

In addition to upper extremity nerve lesions, the impact of PNI has also been studied in patients with lower extremity nerve damage, e.g., peroneal mononeuropathy. Aprile assessed QoL by means of the SF-36 in 69 patients with peroneal mononeuropathy and found significantly lower scores for the aspects of vitality, social function and is emotional role in their study sample as compared to healthy subjects. However, when stratification was performed to exclude patients with peroneal mononeuropathy which reported predisposing factors which were likely to affects QoL, no significant differences between the healthy sample and the sample with peroneal mononeuropathy were found [44]. 

## 4. Psychosocial Factors as Predictors of Functional Outcome Following Treatment of Peripheral Nerve Lesions

### 4.1. Surgical Repair of Traumatic Nerve Injuries

Hundepool et al. conducted a prospective multicenter study in 61 patients, aiming to identify prognostic factors for functional recovery in the first postoperative year following injuries of the median nerve (*n* = 28), ulnar nerve (*n* = 27) or combined lesions (*n* = 6) at forearm level. The majority of patients (85%) were blue-collar workers and the median educational score equaled a high-school degree. One year after injury, 84.6% of patients had returned to work. Besides the identification of the DASH score, power grip and sensibility of the hand as best prognostic factors, the aforementioned authors found gender, level of education as well as posttraumatic levels of stress at one- and three months post-injury as highly predictive in regard to functional recovery [45]. Building on the aforementioned authors’ work, Goswami and colleagues evaluated ten patients with isolated or combined transection lesions of the median and ulnar nerve three weeks and approximately one year following surgical treatment of their injuries, aiming to identify potential predictors for the observed functional outcome. The patients completed the Brief Pain Inventory (BPI) Short Form, NEO Five Factor Inventory (NEO-FFI), and Pain Catastrophizing Scale (PCS) and the McGill Pain Questionnaire (MPQ) at both postoperative time points. Ten healthy individuals served as control group and were evaluated by means of the NEO and PCS. The authors found that pain-catastrophizing was correlated both with the reported pain intensity as well as the occurrence of neuropathic pain. The level of pain-catastrophizing at the first postoperative time-point served as predictor for cold pain thresholds twelve months postoperatively. The level of chronic pain reported at the second assessment time point was also related to the level of pain-catastrophizing as assessed by the PCS which in turn showed correlation with cold pain threshold at this time point [46]. Logically related to Goswami’s research question, Mackinnon’s group investigated the relationship between psychosocial factors and pain relief following peripheral nerve surgery. 331 patients who underwent surgery for peripheral nerve injuries or compression neuropathies and returned for at least two postoperative follow-ups were included. On the one hand, an increased impact of pain on QoL or reported anger, respectively were significant predictors of next-visit pain. On the other hand, self-reported hopefulness, sadness, and depression were not found to be predictive of next-visit pain. Patients who suffered from upper extremity PNI and refused to comment on a possible history of childhood trauma had a significant association with both same-visit pain and next-visit pain. The level of pain served a significant predictor of the reported impact of pain on QoL, sadness, depression, anger, and hopefulness during the next visit. Lower extremity nerve injury was predictive of anger during the next visit, whereas upper extremity nerve injury had no predictive value. Female sex served as a significant predictor for next-visit sadness and anger. Next-visit sadness and depression could be predicted in case the patient reported a positive history of childhood trauma, which was the case in 7.9% of study sample. While 89.3% of the patients denied childhood trauma, 2.8% refused to comment on this question [47]. In conclusion, the listed studies’ results suggest that surgeons should be aware of the fact that functional recovery following repair of peripheral nerve lesions can be significantly influenced by the prevalence of postoperative stress and pain-catastrophizing. In regard to the psychosocial Sadness and depression, although not predictive of functional outcome in the limited number of studies investigating this relationship, are more likely in patients suffering from PNI and have a positive or suspected positive history of childhood trauma.

### 4.2. Surgical Treatment of Compression Neuropathies

Besides cases of traumatic nerve injuries, patients suffering from compression neuropathies also frequently require surgical treatment. In consequence, predictors of functional outcomes following peripheral nerve decompression have been studies by several groups. A retrospective study aiming to identify outcomes of care and predictors of disability and health status in adults with peripheral nerve injuries included >360 patients with PNI which underwent surgical treatment. Included patients presented with 1. median nerve compression; 2. ulnar neuropathy; 3. mixed median and ulnar nerve compression; 4. radial nerve palsy; 5. thoracic outlet syndrome or 6. brachial plexus injury. About 80% of the patients were treated operatively and 70% of these underwent nerve release while the remaining 20% received conservative treatment. The authors found that while health status changed minimally, significant improvements in disability, work disability, pain, depression, and stress were observable following any treatment. At discharge, 57% of employed patients had resumed their work. No significant differences were observable between patients who were treated surgically or those who underwent conservative therapy. Disability was most significantly increased in patients with brachial plexus injuries. More favorable outcomes were observable in patients who pursued gainful employment and had reported symptoms less than six months prior to treatment. Post-treatment functional outcomes could be predicted by psychosocial factors like work status, household management, pain, depression, stress, and difficulty sleeping [48]. In addition to Stonner’s comprehensive analysis of patients with different compression neuropathies, other authors have evaluated cohorts of patients suffering from one distinctive nerve compression syndrome alone with the results summarized in the following paragraphs.

#### 4.2.1. Peroneal Nerve Decompression

In 2019, Wilson et al. published the results of a retrospective study aiming to identify potential predictors of functional outcome following peroneal nerve decompression at the level of the fibular head. The working status of the included patients was also evaluated in regard to a possible correlation with functional outcome. However, no statistically significant correlation could be identified [49]. 

#### 4.2.2. Ulnar Nerve Decompression

Gaspar’s group aimed to evaluate predictors for revision surgery both following in situ ulnar nerve decompression [50] as well as medial epicondylectomy [51] in patients with cubital tunnel syndrome. While revision surgery following in situ decompression of the ulnar nerve was required in 3.2% of the analyzed 216 cases, patient age < 50 years was the only significant predictor of revision surgery. Neither gender nor workers ‘compensation status had any predictive significance. In regard to patients who underwent medial epicondylectomy, 13.3% of the 82 cases required revision surgery. In accordance with the aforementioned study, younger age was identified as predictive factor. Workers’ compensation claims, lesser disease severity, and preoperative opioid use were identified as additional significant predictors. 

#### 4.2.3. Treatment of Carpal Tunnel Syndrome

In 2017, Jerosch-Herold and colleagues published the results of large multicenter study aiming to identify prognostics factors for functional outcome and resulting costs for carpal tunnel syndrome either treated by surgical decompression or corticosteroid injection. They found a highly significant correlation between the patient-reported carpal tunnel symptom severity, depression, anxiety and the health-related QoL as assessed by the EQ-5D-3L (3-level version of EuroQol-5 dimension). The level of anxiety was also correlated with the objective carpal tunnel syndrome severity as assessed by electrophysiological evaluations, but there was no correlation of electrophysiological findings and depression [52]. Straub’s group evaluated 100 hands of 67 patients, respectively, who underwent endoscopic carpal tunnel release, aiming to identify patient- and psychosocial factors associated with unsatisfactory outcomes. Out of the 8% percent of hands which were classified as unsatisfactory 75% were covered by the worker’s compensatory system and in 21% of cases which were involved in litigation an unsatisfactory result was reported. Interestingly, the author concluded that various comorbid factors which were assessed, e.g., obesity, smoking or working in a job at risk did neither in isolation nor in combination result in an increased likelihood for unsatisfactory results. However, psychological factors, e.g., use of psychotropic medications or active psychiatric treatment, which were found positive in 20% of the study population, were associated with lower patient satisfaction both in isolation and combination [53]. In 2008, Lozano Calderón and colleagues conducted a retrospective survey to evaluate patient satisfaction following open carpal tunnel release and included 82 participants. They found that greater levels of depression were associated with more severe dissatisfaction following surgery and perceived disability could be predicted by depression and pain catastrophizing. The authors concluded that depression and perceived disability after carpal tunnel release can be predicted primarily by psychosocial factors like depression and insufficient coping skills [54]. Das De et al. found a significant correlation between DASH scores of patients suffering from carpal tunnel syndrome and psychosocial factors such as depression, catastrophic thinking, kinesiophobia and a punishing response by the respective patient’s partner. Conversely, pain anxiety as well as solicitous or distracting responses by the respective patient’s partner did not correlate with disabilities of the upper extremity in patients with carpal tunnel syndrome [55]. To summarize, these studies indicate that disease severity in patients with carpal tunnel syndrome is often directly linked to psychosocial aspects of life on the one hand whereas patients suffering from depression or anxiety are likely to experience adverse functional outcomes following carpal tunnel release. 

### 4.3. Recovery of Donor Site Morbidity following Nerve Harvest

Ehretsman and colleagues evaluated subjective healing of nerve donor site morbidity following nerve graft harvest by means of a telephone survey. The authors evaluated possible correlations between satisfaction with donor site morbidity, both in regard to functional and cosmetic factors, and patient factors such as age, gender, involvement of worker’s compensation and/or ongoing litigation. However, no statistically significant correlation with patient factors was observable [56]. Another study conducted by Miloro’s group assessed patient satisfaction following sural nerve harvesting in 47 patients. In accordance with Ehretsman’s results these authors did not observe any correlation between patient factor like age, gender and legal involvement and satisfaction level in regard to the donor site [57]. 

### 4.4. Treatment of Painful Neuroma

Stokvis et al. conducted a literature review aiming to identify possible prognostics factors for insufficient pain relief following neuroma treatment in 2009 [58]. They extracted ongoing worker’s compensation, employment status and active litigation as predictive factors for unsuccessful treatment attempts. However, the authors stated that these factors are very difficult to consider separately, given the fact that employment status is likely more important regarding the outcome of patients undergoing surgery for painful neuroma [59,60]. Stovkis’ group also emphasized Dellon’s and Mackinnon’s observation, that the duration of preoperative pain was significantly longer in patients who reported poor pain relief following surgery when compared to patients with satisfactory postoperative amelioration of pain [58,60]. In 2019, Lans and colleagues retrospectively analyzed 33 painful neuromas in 29 patients who underwent surgical therapy. Comparing the three treatment concepts of 1. neuroma excision with consecutive nerve repair or reconstruction; 2. neuroma excision with implantation of the proximal stump and 3. neuroma excision alone. In their study population the mean PROMIS Upper Extremity score was 45.2 ± 11.2, the mean PROMIS Pain Interference score was 54.3 ± 10.7, and the median numeric rating scale pain score was 3 (interquartile range, 1 to 5). Higher PROMIS depression scores and the surgical concept of neuroma excision alone were both independently significantly correlated with lower PROMIS Upper Extremity scores. Postoperative PROMIS Upper Extremity scores were lower in patients who underwent neuroma excision with nerve stump implantation, but this was not statistically significant. Neuroma excision alone and neuroma excision with nerve stump implantation as well as higher PROMIS Depression scores were all independently associated with higher, e.g., more severe PROMIS Pain Interference scores. Higher numeric rating scale pain scores showed a significant correlation with neuroma excision alone and neuroma excision and implantation whereas neuroma excision with consecutive nerve repair or reconstruction was associated with lower numeric rating scale pain scores [61]. 

## 5. A Perspective on Experimental Insights

In addition to clinical studies, psychosocial aspects of peripheral nerve injuries have also been studied in preclinical models, e.g., rodents. Using a spared nerve injury (SNI) model Norman et al. tested the hypotheses that peripheral nerve injury is causative for depression by induction of inflammatory processes in the brain and these neuroinflammatory changes are further exacerbated in case of stress exposure prior to nerve injury. The authors found their presumptions to be confirmed as they observed that injury of the common peroneal and tibial nerve caused mechanical allodynia and depressive behavior in mice, as well as an increased expression of interleukin-1b (IL-1b) and glial fibrillary acidic protein (GFAP). The mechanical allodynia was more severe in mice which were exposed to increased stress by chronic physical constraint two weeks prior to the experimental surgery. Treatment of these animals with a corticosteroid synthesis inhibitor prior to physical constraint eliminated the aforementioned effects, proving that psychosocial factors, e.g., the experience of increased stress directly influences the severity of symptoms following peripheral nerve injury [62]. 

Besides individual psychological factors like depression, social factors have also been identified to play an important role in symptom severity in rats with peripheral nerve injury. Raber and Devor used a neuroma model of neuropathic pain caused by sciatic nerve injury to investigate pain phenotype in two distinct rat strains. When rats with high (HA) and low (LA) pain phenotype and autotomy-behavior, e.g., gnawing of the toes or entire paws in consequence to nerve injury, were housed together, LA rats showed high levels of autotomy even when they were familiar with the HA preoperatively. The observed autotomy in LA rats was also independent of the performance of autotomy by the HA rats. Interestingly, even the contact with cage bedding soiled by HA rats was sufficient to induce moderate levels of autotomy in LA rats even in the absence of HA rats [63]. Another study investigated the effects of ongoing social stress (OSS) on mechanical sensitivity and cold allodynia in a rat model of chronic constriction injury (CCI) of the sciatic nerve. Rats which experienced ongoing social stress by twice-weekly exchange of their cage mates did not display significant changes in mechanical sensitivity. In regard to cold allodynia, rats with CCI and OSS were less susceptible during the early phase of the observation period when compared to rats which underwent CCI surgery only. At later time points however, rats with CCI + OSS were more susceptible to cold stimuli compared to the CCI rats. In addition, in the former group enhanced glial cell activation, pro-inflammatory cytokine expression and higher neurotrophic factor mRNA levels were observable [64].

## 6. Discussion

In this work we reviewed the current body of knowledge in regard to the interplay of psychosocial factors and peripheral nerve lesions as well as these factors’ predictive value of functional outcome following peripheral nerve injury. Our work emphasizes that psychological factors like depression, pain-catastrophizing and anxiety are both influenced by peripheral nerve lesions and also significant predictors of functional recovery and QoL after peripheral nerve surgery in patients suffering from PNI. The same applies to social factors, e.g., employment status or worker’s compensation. These findings underpin the need for personalized treatment concepts involving not only surgeons but also psychologists, occupational therapists, and others. As was pointed out by Kaltenbrunner and other authors [65,66] there are large differences between countries in regard to the regulatory framework of disability cases and rehabilitation measures to facilitate the affected individuals return to work. Notably, this not only applies to the transatlantic comparison, but also within the smaller perimeter of the European Union, indicating the need to consider the country-dependent differences when developing treatment and rehabilitation concepts for patients with PNIs.

Patients with depression, pain catastrophizing and anxiety are usually at risk to experience poor outcomes following PNI, reporting higher levels of pain and disability as well as lower satisfaction [54]. Vice versa, the rate of symptoms of clinical depression among patients suffering from PNI is alarmingly high, reaching almost 40%, which is more than twice the numbers reported in the general population, ranging between 10% and 20%, depending on the studied population [38,67,68]. In case of brachial plexus injuries, even more than 50% of patients could be suffering from depression, underpinning the need for adequate treatment strategies beyond surgical intervention in this group of patients [69,70]. In conclusion, screening for depression and referral of patients for psychological and/or psychiatric counseling or treatment is advised for surgeons and any other profession involved in the treatment of patients with PNIs, especially in case of a planned operative intervention [38]. However, it might be demanding to identify such patients since they might show a tendency to conceal their depression, afraid of the social stigma which might come with diagnosed mental illness [17,71]. Circling back to the aforementioned cross-country differences regarding post-injury rehabilitation and return to work, the same applies to mental health care systems. Again, significant differences are not only observable when comparing mental health care systems worldwide [72] but also within the European Union [73]. These observations emphasize that the interplay of psychosocial factors and peripheral nerve lesions extends beyond the affected patients’ ways of living but are also heavily influenced by significant differences between countries regarding their health care system. 

In our opinion, the findings reported by Ehretsman [56] and Miloro [57] deserve special emphasis, as both authors reported that donor site morbidity following nerve harvest was not correlated with any of the psychosocial factors they assessed. Although nerve graft harvesting can be considered as nerve lesion, it is interesting to note that sequalae of these “non-accidental” nerve injuries seem not to be correlated with psychosocial factors as it is the case with traumatic nerve lesions or compression neuropathies. As possible explanation for this observation we would like to suggest that patients who undergo nerve graft harvest choose this procedure voluntarily and without the experience of a “loss of control” associated with traumatic nerve injuries. It was shown that the feelings of uncontrollability or helplessness are associated with an increase in psychological vulnerabilities [74,75,76] and pain levels [77,78]. The patient’s impression of being in control of the situation leading to a nerve lesion, e.g., sural nerve harvest, might be protective of adverse functional outcome following these procedures. 

Another interesting finding was the correlation between picking “no comment” when asked about a possible childhood trauma and pain reported at the current and next visit. The same applies to the predictive value of a positive history childhood trauma for next-visit sadness and depression. It has been suggested that this correlation is caused by trauma-induced changes to the brain of abused or traumatized children [47,79,80]. In this context, one should consider the fact that about 10% of American youth have experienced at least one episode of sexual assault and 9–19% were subject to physical abuse or a physical assault by the respective caregiver [81]. Although a history of childhood trauma does not necessarily cause pain, sadness and depression, the likelihood of seeing an abused person with PNI at the inpatient or outpatient clinic is relatively high. 

As several studies reviewed in this work have pointed out there are distinct variations regarding the impact of PNIs on psychosocial factors depending on their severity. Patients with distal, single-site compression neuropathies will likely experience fewer negative psychosocial effects that patients with distal traumatic injuries of both the median and ulnar nerve, dual compression neuropathies or TOS. As was emphasized by Wojtkiewicz these findings bear several implications for clinical practice, as patients suffering from the aforementioned conditions should be counseled regarding the impact of such PNIs on their psychosocial and occupational status prior to surgical treatment [17]. The ability to return to work (RTW) is another exemplary psychosocial factor, as it is primarily affected by PNI but also has an impact on functional recovery in patients with PNI. Knowledge of this interplay is of high value when an individual treatment and rehabilitation plan is conceptualized for the patient as patients with more complex injuries, e.g., combined nerve injuries of the upper extremity are at high risk not to return to work. In consequence functional recovery in these patients might also be poor, given their inability to pursue their profession as desired [34]. Considering the exorbitant indirect costs of low productivity which exceed direct health costs by more than 100% in case of upper extremity PNIs [14,82,83,84] an adequate prognostic assessment and a personalized interdisciplinary treatment are of outmost relevance. A battery of structured preoperative assessment tools such as the PROMIS-29 and EQ-5D [85] are suited to determine the impact of peripheral nerve injuries on patient-reported QoL. The healthcare team involved in treatment of patients with PNI should consist of expert not only in surgical treatment of nerve injuries, but also specialist for physical as well as emotional adaptation and resilience, e.g., hand therapists, occupational therapists, psychologist, and social workers [86]. 

Wojtkiewicz summarized the evidence gathered in the literature regarding the influence of pain caused by PNIs on patient-reported disability [17,18,38,87,88,89,90]. Pain levels can be assessed by the BPI Short Form, NEO-FFI, PCS and the MPQ. A more personalized pain-assessment is possible via pain drawings [91]. It was shown that these drawings are affected by pain and depression in patients with cervical degenerative disc disease [92] or cervical spine nerve involvement in chronic whiplash-associated disorders [93]. They are also a feasible and reliable tool to assess neuropathic pain following spinal cord injury [94]. Pain drawings are also predictive of functional outcome in patients undergoing surgical treatment for degenerative disc disease in the cervical spine [95]. Use of a related assessment-tool named CALA to visualize pain in upper limbs amputees has been published by Prahm et al. [96], but there is yet no published large patient sample study evaluating the value of pain drawings in patient with peripheral nerve lesions in general. Given the high prevalence of neuropathic pain of up to 10% in society and its deleterious impact on physical and psychical function [47], a more personalized assessment tool might be a valuable addition to the armamentarium of diagnostic and prognostic instruments.

To summarize our findings, coaching, and providing emotional support to patients suffering from PNI can be effective to help them adapting a positive mindset, overcome severe psychological distress, and eventually adapting to their new situation, even if the functional outcome following surgical treatment is not more than mediocre. It must be emphasized that objective impairment, e.g., severe paresis, or diminished sensibility does not inevitably result in the same level of subjective disability. This observation has been beautifully condensed by Ring who had reconstructed the median nerve in a female nurse following complete iatrogenic laceration: “Credit goes to her (the patient’s) spirit, adaptation and resiliency; not my knife or suture” [86]. 

## 7. Conclusions

Psychosocial factors play an important role in case of PNI. They are not only directly affected by PNI but also have significant predictive value of functional outcome following surgical treatment. Careful psychological assessment can help to identify patients at risk for unsatisfactory functional recovery and persistent disability following surgical treatment. The interplay of psychosocial factors and PNIs should be kept in mind in regard to personalized treatment concepts for these patients. 
